# The role of Xpert MTB/RIF using bronchoalveolar lavage fluid in active screening: insights from a tuberculosis outbreak in a junior school in eastern China

**DOI:** 10.3389/fpubh.2023.1292762

**Published:** 2023-12-22

**Authors:** Qian Wu, Kun-Yang Wu, Yu Zhang, Zheng-Wei Liu, Song-Hua Chen, Xiao-Meng Wang, Jun-Hang Pan, Bin Chen

**Affiliations:** Department of Tuberculosis Control and Prevention, Zhejiang Provincial Center for Disease Control and Prevention, Zhejiang, China

**Keywords:** tuberculosis, outbreak, screening, Xpert MTB/RIF, school

## Abstract

**Background:**

Tuberculosis (TB) outbreaks in schools present a public health challenge. In order to effectively control the spread of transmission, timely screening, accurate diagnosis and comprehensive epidemiological investigations are essential.

**Methods:**

In July 2021, a TB outbreak occurred in a junior high school in Y City, Zhejiang Province. Students and faculty were screened for TB by symptom screening, chest radiography, and tuberculin skin test during four rounds of contact screenings. For sputum smear-negative and sputum-scarce patients, bronchoscopy was used to collect BAL samples for Xpert *Mycobacterium tuberculosis*/rifampin (MTB/RIF). Whole-genome sequencing and bioinformatics analysis were performed on isolates to identify the strains of MTB isolates and predict drug resistance.

**Results:**

Between July 2021 and November 2021, a total of 1,257 students and faculty were screened for TB during screenings. A total of 15 students (1.2% of persons screened) aged 15 years were diagnosed with TB. Eighty percent (12/15) of the cases were laboratory-confirmed (10/12 [83%] Xpert MTB/RIF-positive, 2/12 [17%] culture-positive). Most cases (12/15 [80%]) were in students from Class 2. All cases were asymptomatic except for the index case who had symptoms for more than two months. Seven MTB isolates were collected and belonged to lineage 2.

**Conclusion:**

Our findings demonstrated the potential of Xpert MTB/RIF using BAL as a screening tool in school TB outbreaks for sputum smear-negative and sputum-sparse suspects, which may not only rapidly improves diagnostic accuracy, but also facilitates epidemiological investigations and homology analysis.

## Background

Tuberculosis (TB) is an airborne disease primarily acquired by inhalation of infectious aerosol particles containing *Mycobacterium tuberculosis* (MTB). TB in children and adolescents is a growing concern and problem, especially in countries with a high TB burden (1, 2). However, the decrease in the reported incidence of TB among students decreased at a lower rate than in the whole population, the proportion of reported cases among students has been increasing (3). Thus, the prevention and control of TB in schools is a focus of China’s TB prevention and control programs.

In recent years, TB in children has become a more recognized problem in China largely due to reports of TB outbreaks in schools and households (4–7). In addition to greater susceptibility among schoolchildren (2, 8), crowded classrooms predispose to TB transmission in schools. However, an important issue that impedes case detection in pediatric TB is the high proportion of children with TB that are unable to provide sufficient sputum or have negative smear sputum (9–11). However, microbiological confirmation of TB is possible only in a small minority of the students, and the time to diagnosis by sputum culture is often prolonged (12). Alternative measures to improve case detection among children are urgently needed. Bronchoscopy not only allows observation of trachea and bronchial lesions, but also allows for the collection of bronchoalveolar lavage fluid (BAL) samples. For sputum scarce/negative patients, bronchoscopy may be a safe and reliable method for collecting of eligible samples (13). Rapid diagnosis and transmission investigation are key to effectively controlling the spread of school TB outbreaks. However, diagnosis is challenging when a patient is clinically and radiologically suspected of having TB but sputum specimens lack laboratory-confirmed evidence. Xpert *Mycobacterium tuberculosis*/rifampicin (MTB/RIF) assay is an automated nucleic acid amplification test that is recommended by World Health Organization (WHO) as the initial diagnostic test for patients presumed to have TB on the basis of screening symptoms or chest radiography, or for the diagnosis of multidrug-resistant disease (14). This molecular method performs well and can even confirm the diagnosis even from paucibacillary, mucoid and salivary sputum.

On July 2021, a TB outbreak occurred in a junior high school in Y City, Zhejiang Province. We investigated the potential role of Xpert MTB/RIF using BAL samples for the diagnosis of sputum smear-negative and sputum-scarce TB in active screening in order to provide a scientific basis for future investigations and management of TB outbreaks in schools.

## Methods

### Study setting

The school has 79 classes from first grade to ninth grade, with more than 3,600 students and 260 staff. Of these students, 222 are residential, with 4–8 students per dormitory. The dormitories and classrooms are well ventilated. The school clinic is equipped with a full-time doctor. However, health supervision measures in schools, such as the records of the morning, afternoon and evening health check-ups of students, were irregular or incomplete. In addition, tracing and registration records of absence due to illness were incomplete.

An investigation was conducted on each case, including collecting general information (such as name, sex, age, grade, class, seat distribution, close contacts), and information on epidemiological investigations, and clinical diagnosis and treatment. Reports on the status of the outbreak, including the epidemiological characteristics, causes, prevention and control measures were updated regularly until the end of the outbreak. In this study, we used a descriptive epidemiological approach was to retrospectively analyze data from the questionnaire of cases and the report of epidemic disposal.

#### Diagnosis and definitions

All TB cases, including laboratory-confirmed cases and clinically diagnosed cases, were defined according to the Diagnosis for Pulmonary Tuberculosis (WS288-2017) (15). Due to the inability of children to voluntarily expectorate sputum, microbiological confirmation of TB in children is complicated. Bronchoscopy was performed for suspected patients with negative sputum smears or without eligible sputum specimens. Children are considered to be suspected TB patients if their chest radiography shows lesions consistent with active TB. All suspected TB patients who underwent bronchoscopy provided written informed consent. Xpert MTB/RIF were performed, which is recommended by the World Health Organization (16). Compared to microscopy, Xpert MTB/RIF helps to provide rapid confirmation of TB and has a higher sensitivity for diagnosing TB in a population of children aged 0–15 years (17). Laboratory-confirmed cases were defined as those with MTB present in sputum smear or sputum culture or Xpert MTB/RIF.

Clinically diagnosed TB cases were presumptive TB cases with negative sputum smear microscopy that met the following conditions: (1) other lung diseases were excluded after diagnostic treatment or follow-up observation, (2) a chest radiography that was indicative of TB accompanied by TB-related symptoms (e.g., chronic cough, fever, night sweats, weight loss) or a strongly positive tuberculin skin test (TST) result.

An outbreak of aggregated MTB infection is identified when two or more cases of epidemiologically linked active TB (including TB pleurisy) are diagnosed in a school within a period of 3 months. When 10 or more epidemiologically linked cases of TB or fatal cases of TB in the same semester, the health administration department assigned to the school decides whether to declare a public health emergency (18, 19).

The first case of active TB reported in the school was defined as the index case. Depending on the mode, degree and duration of contact with the index case, contacts classified as close contacts, general contacts, and occasional contacts. Students who studied or lived with someone with active TB for a long time were classified as close contacts. Students who studied on the same teaching floor or lived on the same dormitory floor as the index case were classified as general contacts. Students who studied in the same teaching building or lived in dormitory building, but not on the same floor as the index case were classified as occasional contacts.

#### Contact screening

Four rounds of contact screening were performed, which included symptom screening, TST, chest radiography, sputum smear microscopy, sputum culture, bronchoscopy and Xpert MTB/RIF. TST was performed by injecting 0.1 mL (5 IU) of purified protein derivative of tuberculin under the skin of the left forearm on the palmar side and measuring the size of the induration after 72 h. TST reactions with a diameter of induration was ≥15 mm and/or in the presence of blisters, necrosis or lymphangitis were defined as strongly positive. Those with a strongly positive TST or abnormal chest radiography result were examined by computed tomography (CT), bronchoscopy and Xpert MTB/RIF. BAL sample was harvested from each patient by doctors of First People’s Hospital of Y City. BAL sample was mixed with pretreatment reagent of the Xpert MTB/RIF (Cepheid, United States) for 15 min, then 2 mL pretreated sample was transferred to the Xpert MTB/RIF (Cepheid, United States) cartridge and test program was executed. Sputum smear microscopy scoring of positivity was in accordance with the standard of WHO (20). Culture medium was Löwenstein-Jensen medium (Baso, Zhuhai, China) and positivity scored followed the standard of WHO (21).

#### Genome sequencing

Genomic DNA was extracted by the SDS method for *M. tuberculosis* (22). The harvested DNA was detected by agarose gel electrophoresis and quantified using a Qubit 2.0 Fluorometer (Thermo Fisher Scientific, Waltham, MA, United States). A total of 0.25 μg DNA per sample was used as input material for the DNA sample preparations. Sequencing libraries were generated using NEBNext Ultra DNA Library Prep Kit for Illumina (New England Biolabs, Inc., Ipswich, MA, United States). Briefly, the DNA sample was fragmented by sonication to a size of 350 bp, then the DNA fragments were end-polished, A-tailed, and ligated with the full-length adaptor for Illumina sequencing with further PCR amplification. Finally, PCR products were purified using AMPure XP system (Beckman Coulter, Brea, CA, United States) and libraries were analyzed for size distribution using an Agilent 2,100 Bioanalyzer (Agilent Technologies, Santa Clara, CA, United States), and quantified using real-time PCR. Whole-genome sequencing was performed using Illumina NovaSeq PE150 (Beijing Novogene Bioinformatics Technology Co., Ltd., Beijing, China) with an estimated mean coverage of 100 times.

The raw data were filtered on the basis of these steps: the reads originated from the host, the reads containing low-quality bases (mass value ≤20) over a certain percentage (default: 40%), the number of N in reads beyond a certain proportion (default: 10%), and the reads, overlap between them and the adapter exceeded a certain threshold (default: 15 bp) with less than 3 mismatches. Samples that met these conditions were removed. The clean data were assembled simultaneously using SOAPdenovo software (BGI Genomics, Shenzhen, China), SPAdes software (University of California San Diego, San Diego, CA, United States) and Abyss software^1^ (23). The assembly results of these three software types were integrated with CISA software (Cybersecurity and Infrastructure Security Agency, Washington, DC, United States) and results containing the least scaffolds was selected. Gapcloser software (BGI Genomics, Shenzhen, China) was used to fill the gaps in the preliminary assembly results. The same lane pollution was removed by filtering the reads with low sequencing depth (less than 0.35 of the average depth) and fragments below 500 bp were filtered out to obtain the final assembly result.

#### Bioinformatics analysis

Tuberculosis-Profiler software (London School of Tropical Medicine and Hygiene, London, United Kingdom) (24) was used to determine isolates lineage and predict drug resistance. Paired-end reads were mapped to the reference genome sequence of H37Rv (GenBank AL123456) with Burrows-Wheeler Aligner software,^2^ and SAMtools/BCFtools suite^3^ was used for calling fixed single-nucleotide polymorphisms (SNPs) (frequency ≥ 95%) (25). The fixed SNPs which were excluded in the PE/PPE guanine-cytosine (GC)-rich sequence and repetitive region were concatenated into one sequence for the phylogenetic tree construction (26). The phylogenetic tree was constructed by Randomized Axelerated Maximum Likelihood (RAxML) software^4^ using the maximum-likelihood method (27), and visualized using ggtree toolkit of R statistical analysis software (R Foundation for Statistical Computing, Vienna, Austria).

## Results

### Basic characteristics

**Figure 1:**
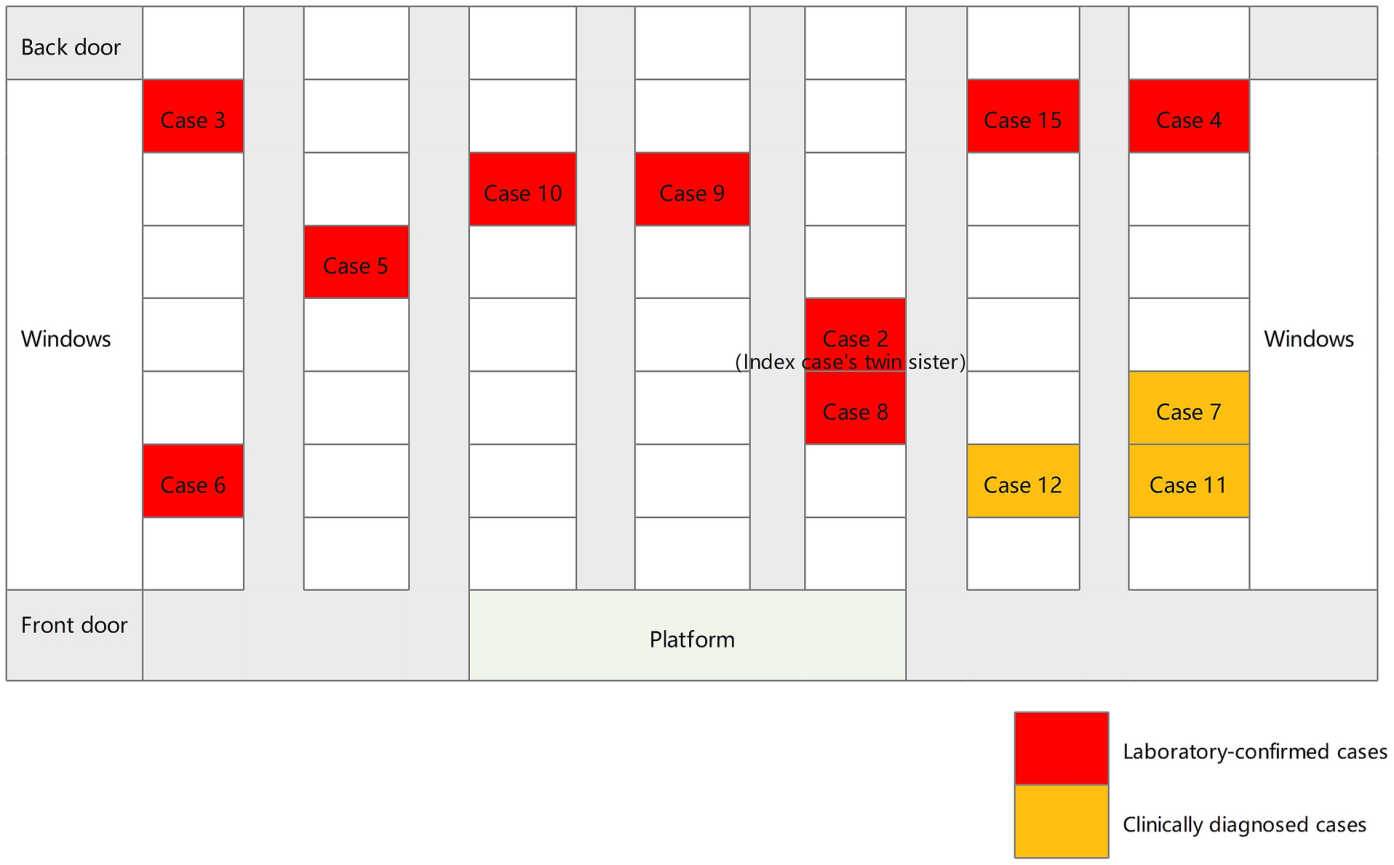
Seating distribution of the students with tuberculosis in class 2.

Between July 2021 and November 2021, a total of 15 students were diagnosed with TB, all of whom were in Grade 9. The average age was 15 years (Table 1). There were nine boys and six girls. Cases 1–6, 8–10, 13–15 (80%) were laboratory-confirmed, 2 (cases 2 and 15) of whom were sputum culture-positive and 10 (cases 1, 3–6, 8–10, 13–14) were Xpert MTB/RIF-positive. Cases 7, 11 and 12 (20%) were clinically diagnosed. The index case developed initial TB symptoms in April 2021 and the diagnosis was confirmed by Xpert MTB/RIF on July 2. Apart from the index case, all other cases were clinically asymptomatic. On August 10, the twin sister of the index case (case 2) was sputum culture positive. In terms of class distribution, there were 12 cases of TB in Class 2, and 2 cases in Class 1, and 1 case in Class 3. The seating distribution of the cases in Class 2 is shown in Figure 1. Except for case 9, all students diagnosed with TB were non-resident students.

**Table 1:** The demographic and baseline characteristics of the students with TB.

No.	Sex	Age	Grade	Class	Suspicious symptoms	TST	Chest radiography	CT	Methods and results	Date of diagnosis	Treatment
1	F	15	9	1	Yes	-	Abnormal	Abnormal	Sputum smear(−),Xpert(+)	July 2, 2021	2HREZ/10HR
2	F	15	9	2	No	Positive	Normal	Abnormal	Sputum smear(−),Sputum culture(+),Xpert(−)	August 10, 2021	2HRZE/4HR
3	M	15	9	2	No	-	Abnormal	Abnormal	Sputum smear (−),Xpert(+)	August 26, 2021	2HREZ/10HR
4	M	15	9	2	No	Positive	Normal	Abnormal	Sputum smear(−),Xpert(+)	August 26, 2021	2HREZ/10HR
5	M	15	9	2	No	Positive	Abnormal	Abnormal	Sputum smear(−),Xpert(+)	August 27, 2021	2HRZE/4HR
6	M	15	9	2	No	-	Abnormal	Abnormal	Sputum smear(−),Xpert(+)	August 29, 2021	2HREZ/10HR
7	M	15	9	2	No	Positive	Abnormal	Abnormal	Sputum smear(−),Sputum culture(−),Xpert(−)	August 29, 2021	2HRftEZ/4HRft
8	F	15	9	2	No	-	Abnormal	Abnormal	Sputum smear(−),Xpert(+)	August 30, 2021	2HRZE/4HR
9	M	15	9	2	No	-	Abnormal	Abnormal	Sputum smear(−),Xpert(+)	August 30, 2021	2HRZE/10HRE
10	M	15	9	2	No	-	Abnormal	Abnormal	Sputum smear(−),Xpert(+)	August 30, 2021	2HRZE/4HR
11	F	15	9	2	No	-	Abnormal	Abnormal	Sputum smear(−), Sputum culture(−),Xpert(−)	September 2, 2021	2HRZE/4HR
12	F	15	9	2	No	Positive	Normal	Abnormal	Sputum smear(−),Sputum culture(−),Xpert(−)	September 2, 2021	2HREZ/10HR
13	F	15	9	1	No	Positive	Abnormal	Abnormal	Sputum smear(+),Xpert(+)	September 6, 2021	2HRZE/4HR
14	M	15	9	3	No	Positive	Abnormal	Abnormal	Sputum smear(−),Xpert(+)	September 20, 2021	2HRZE/4HR
15	M	15	9	2	No	Positive	Abnormal	Abnormal	Sputum smear(−),Sputum culture(+),Xpert(−)	October 18, 2021	2HRZE/4HR

TB: tuberculosis; No.: number; TST: tuberculin skin test; CT: computed tomography; Xpert: Xpert MTB/RIF; (−): negative; (+): positive; H: isoniazid; R: rifampicin; E: ethambutol; Z: pyrazinamide; Rft: rifapentine.

### Diagnosis of the index case

The index case was a 15-year-old girl in the local middle school, who developed a persistent cough and expectoration on April 28, 2021. A month later, she took oral anti-inflammatory drugs for more than 10 days, without any significant relief of her symptoms. She stopped treatment on June 12 due to the need to prepare for the midterm exam. After the midterm exam on June 19, she visited the First People’s Hospital of Y City. The chest radiography showed abnormalities in both lungs. From June 19 to June 28, she was hospitalized and given symptomatic treatment such as anti-inflammatory and mucolytics and tested positive on T-SPOT. On June 29, she was hospitalized in a grade-A tertiary hospital of Z City. Bronchoscopy showed white necrotic material covering the mucosa of the opening of the anterior basal segment of the left lower lobe, and Next Generation Sequencing (NGS) showed that BAL was positive for MTB. On July 2, she was transferred to the designated hospital of Z City. During hospitalization, diagnosis of TB was confirmed by Xpert MTB/RIF.

### Four sequential contact screenings

**Figure 2:**
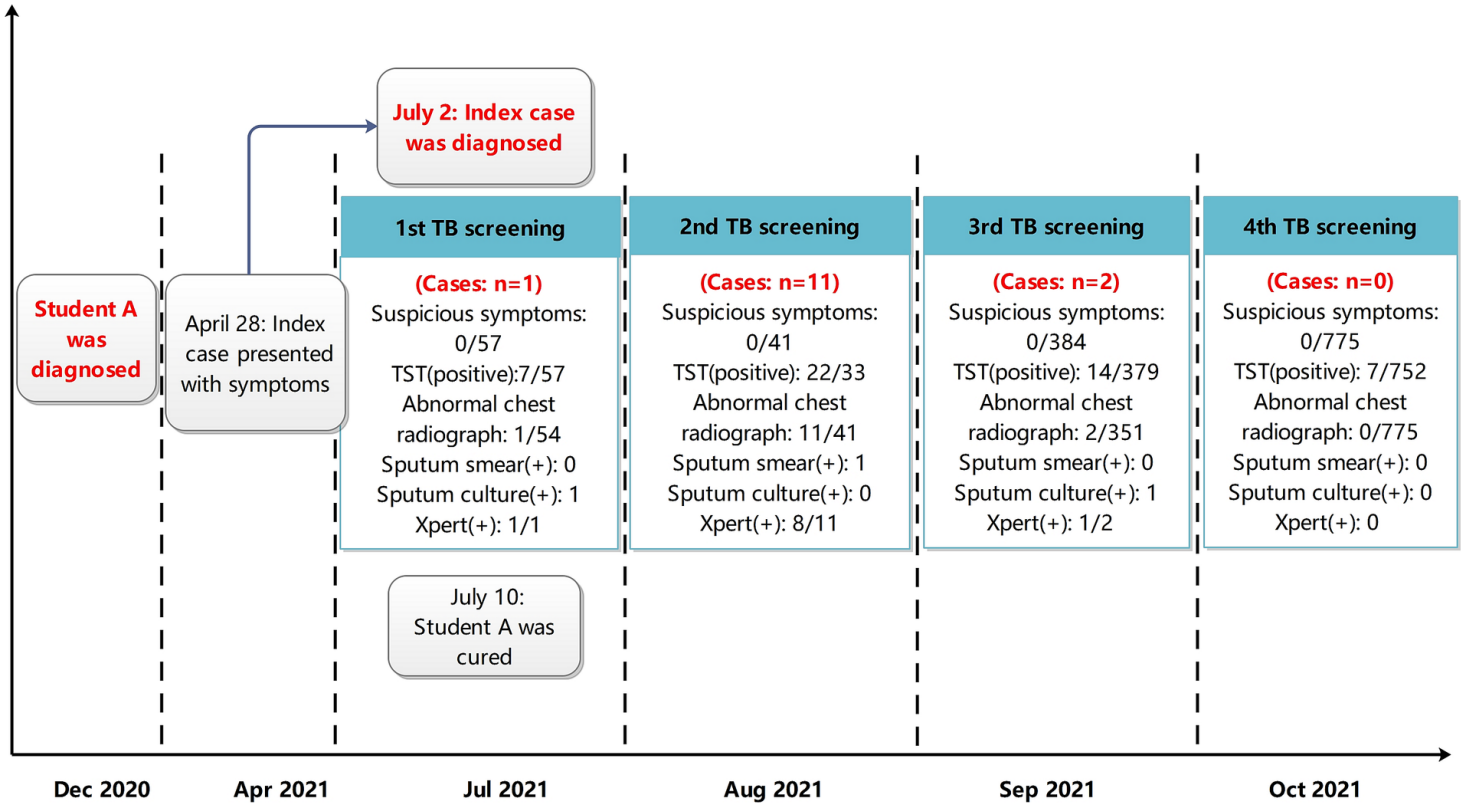
Timeline of the four tuberculosis screenings in a junior high school in Zhejiang, China.

Four rounds of contact screening were carried out among close contacts, general contacts, and occasional contacts of the index case by November 15, 2021 (Figure 2). A total of 1,257 students and the faculty members were screened.

In the first round of screening, starting on July 7, 2021, a total of 57 close contacts of the index case were identified by epidemiological investigation. These included 45 classmates, two students in the neighboring class (Class 2), six teachers, and four family members of the index. All contacts were screened for symptoms, underwent TST and chest radiography. None of the close contacts had any TB-related or any abnormalities on chest radiography. However, seven close contacts (12%) had positive TST results (including 2 in Class 1, 2 in the Class 2, 1 family member, and 2 teachers).

Before receiving TB preventive treament (TPT), the twin sister of the index case developed abnormal shadows on her lungs based on chest radiography. On August 10, her sputum culture was positive and she was reported as a laboratory-confirmed case of TB by the First People’s Hospital of Y City.

In the second round of screening, beginning on August 17, 2021, 41 students in Class 2 were screened for symptoms and received TST and chest X-rays. No students had suspicious TB-related symptoms; however, eleven students had abnormal chest X-rays. Of the 33 students tested for TST, 22 (67%) were positive. A total of 11 confirmed cases were reported, of which eight were Xpert MTB/RIF-positive.

In the third round of screening, starting on August 28, 2021, all students in Classes 3–8, students studying on the same floor, teachers and family members of previously diagnosed cases of TB were screened. None had any suspicious symptoms of TB. The results of TST showed that 14 were positive (3.7%, 14/379). Two students had abnormalities on chest X-ray and a total of two confirmed cases were reported. Case 14 was Xpert MTB/RIF-positive and case 15 was culture-positive.

Starting on September 26, 731 students and 44 teachers from the original 7th and 8th grades were screened. Seven (7/752, 0.9%) were positive on the TST. None of them had abnormalities on chest radiography. Further chest radiography and pathological investigations were performed on those who were TST-positive, and no additional cases of TB were found.

### Bioinformatics analysis results

**Figure 3:**
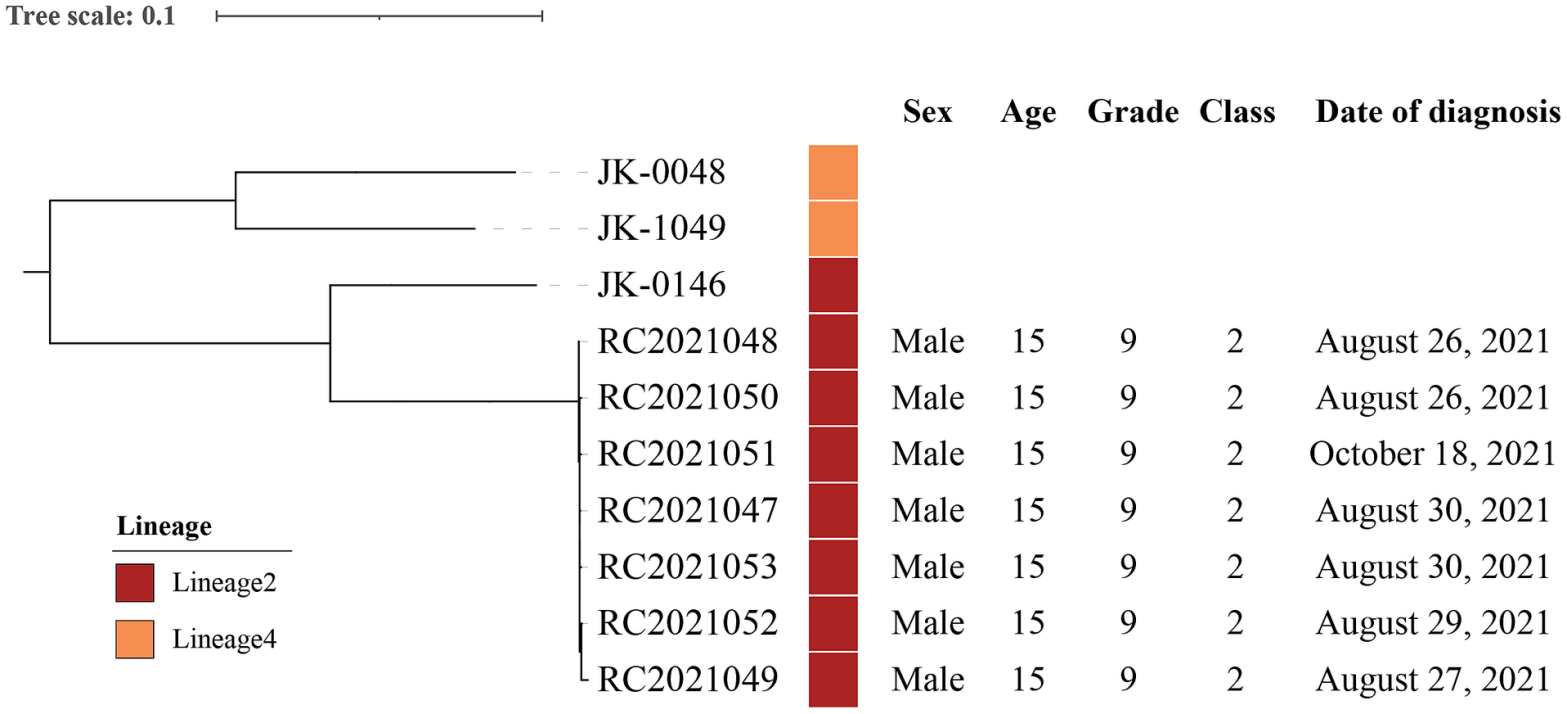
Phylogenetic tree of the *Mycobacterium tuberculosis* isolates from the outbreak.

In order to identify the lineages, genetic relationship, and predict drug resistance, isolates from seven cases (cases 3–6, 9–10, and 15) were analyzed and compared with other clinical strains from 3 laboratory-confirmed TB cases in bioinformatics analysis. A maximum-likelihood phylogenetic tree was constructed using RAxML software and *Mycobacterium canettii* (CIPT 140060008) was at the root of the tree (Figure 3). Two strains belonged to Lineage 4 and the other strains belonged to Lineage 2. Strains (RC2021047, RC2021048, RC2021049, RC2021050, RC2021051, RC2021052, RC2021053) involved in the outbreak were predicted drug-sensitivity profiles based on genomic sequences, and that were in accordance with the result of drug-sensitivity tests. The genomic differences among these strains ranged from 0 to 1,166 SNPs. However, the differences between the strains of this outbreak did not exceed 12 SNPs, which revealed that 7 strains isolated from 7 patients in Class 2 were highly homologous and within the same transmission chain (11). Therefore, the phylogenetic tree showed that 7 strains of this outbreak belonged to the same genomic cluster, which was different from the 3 strains of other unrelated patients. Whole gene sequencing data of strains are deposited in National Microbiology Data Center (NMDC) with accession numbers NMDC40049901 (https://nmdc.cn/resource/genomics/sra/detail/NMDC40049901) and NMDC40049902 (https://nmdc.cn/resource/genomics/sra/detail/NMDC40049902).

### Putative transmission relationship

**Figure 4:**
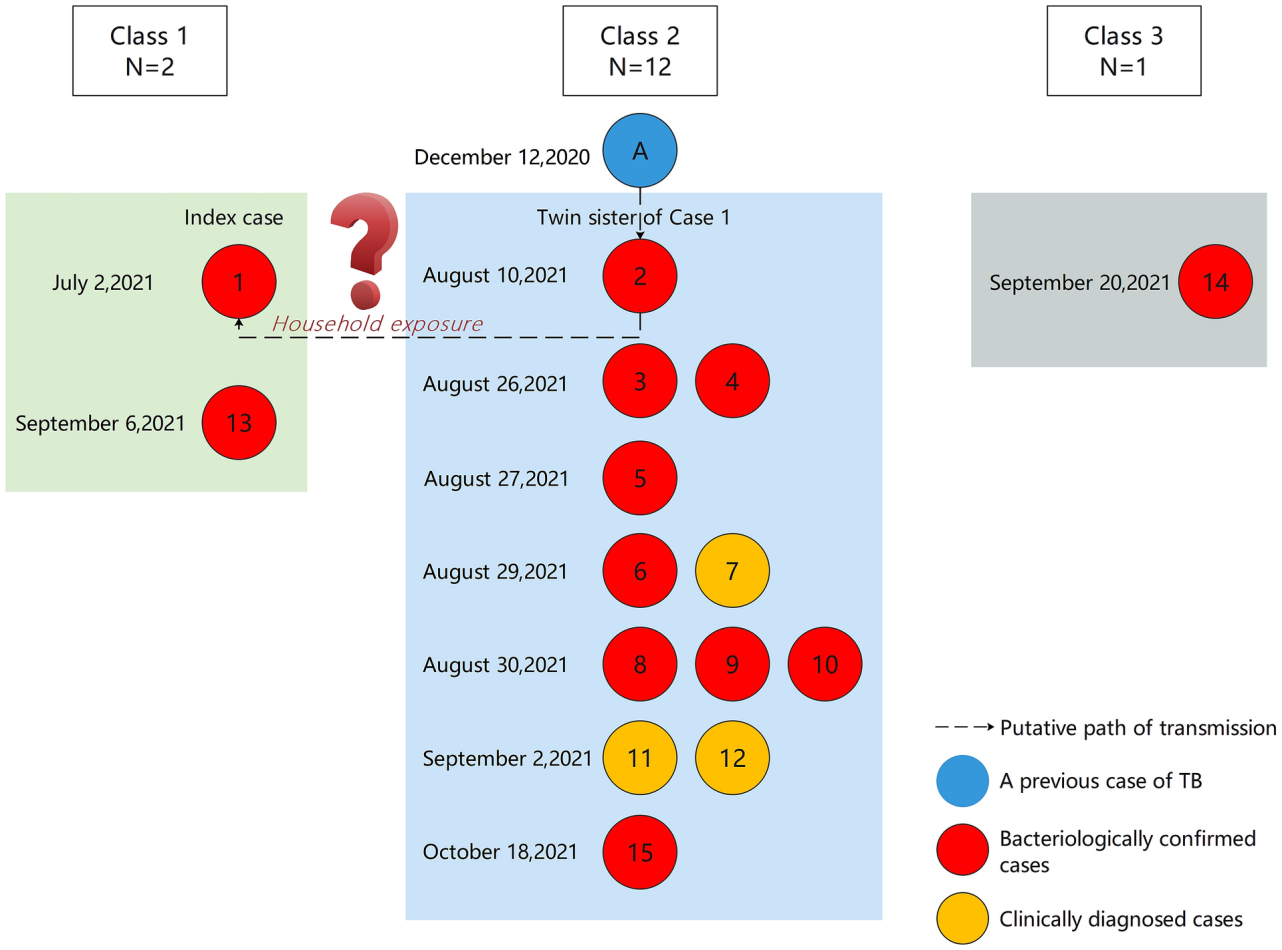
Putative transmission pathways based on epidemiological links.

We found that a student in Class 2, Grade 9 (Student A) had been diagnosed with laboratory-confirmed TB in December 2020. The result of sputum smear microscopy was strongly positive (++++). On July 10, 2021, the student was considered cured and stopped taking medication. Therefore, we hypothesize that student A caused her classmate (case 2) to become infected with TB through excretion of MTB. Although case 2 did not show any symptoms, she may eventually infected her twin sister, the index case, through household exposure (Figure 4).

### Follow-up monitoring

All 15 students with TB were suspended from school after being diagnosed with TB. Follow-up visits confirmed that the majority of patients had a treatment regimen of 2HRZE/4HR or 2HREZ/10HR (Table 1). Liver and kidney function tests were performed regularly for all treated students. No patient experienced adverse effects throughout the course of treatment. Of the 42 students with strong positive TST results during screening, 22 received TPT and the remaining 20 refused TPT. One of those who received TPT had a treatment regimen of 4H, and the remaining 21 students had a treatment regimen of 3HR. Students who refused to take TPT had chest X-rays at the end of the third month, the end of the sixth month, and the end of the twelfth month after the initial contact screening. During the follow-up period, none of these students with positive TSTs developed TB.

## Discussion

An outbreak of TB was reported in a junior high school in Zhejiang, China. Based on the results of the epidemiological investigation, clinical data and laboratory test, we specifically investigated the possible causes of the TB outbreak and determined the likely source and path of transmission.

The lack of awareness of the clinicians who initially treated the index case led to delays in the diagnosis of TB. The clinical manifestations of TB are easily confused with those of other lung diseases (28–30), and the index case diagnosed with TB in a designated hospital more than 2 months after the onset of clinical symptoms. Irregular or incomplete health supervision measures in schools, such as morning (afternoon) and evening health-checks, tracing and registration records of absence due to illness, may also have contributed to students with suspicious symptoms not being adequately monitored (6). Other than the index case, none of students diagnosed with TB, had any suspicious symptoms, which increased the difficulty of case finding. As the outbreak coincided with the run-up to the high school entrance exam, students were under great burden, which may have led to a reduction in their physical resistance and an increased the risk of disease. In addition, junior high school students have inadequate nutrition which may also be a trigger for TB disease progression (4). A reluctance to seek medical treatment also increases the difficulty of early detection. Emerging evidence has shown that delays in seeking care and diagnosis makes it possible for other students to be exposed to pathogens for a long period of time, which is a major contributor to TB transmission (31–33).

In this outbreak, all the TB patients belonged to a single transmission chain. The MTB strains from 7 cases in Class 2 were of the Beijing genotype and were all drug-sensitive, suggesting recent transmission between the students in the cluster of cases.

There are several features worth considering in this school-clustered TB outbreak. It has been reported that subclinical TB can be caused by viable MTB that does not cause any suspicious symptoms of TB (34). Misdiagnosis and delayed treatment of TB, especially sputum smear-negative TB, promotes pathogen transmission (35). However, it may be difficult to detect MTB by conventional testing methods for suspected patients with scarce sputum or sputum smear negativity, especially children (36, 37). It has been shown that the application of bronchoscopic techniques plays an important role in the early diagnosis of cases and in investigating the transmission of TB outbreaks (38). According to a previous meta-analysis, the use of Xpert MTB/RIF for the diagnosis of TB using BAL samples showed high sensitivity and specificity (39). However, studies on the combined use of bronchoscopy and Xpert MTB/RIF in TB outbreaks in schools are less common.

In order to verify cases and trace homologues as quickly as possible in the management of this TB outbreak, all suspected cases agreed to the use of bronchoscopy to collect BAL samples for Xpert MTB/RIF test. As a result, the percentage of laboratory-confirmed cases was high (80%). It is worth pointing out that the clinical application of bronchoscopy and Xpert MTB/RIF is limited to hospitals. The main reason for this is that these techniques rely on relatively complex instruments that are costly. In addition, the collection of BAL samples using bronchoscopy requires invasive procedures.

As many studies have reported, high schools and colleges are among the most common sites of TB outbreaks (4, 6, 26, 32). Although this TB outbreak occurred in a junior high school, these students were about to become high school students. Disposal measures such as contact screening, accurate diagnosis and comprehensive epidemiological investigation effectively avoided the spread of the TB outbreak.

There are several strengths to this study. With the early application of bronchoscopy, the percentage of laboratory-confirmed cases in this school TB outbreak increased considerably. Seven isolates were collected from laboratory-confirmed TB patients, which helps to analyze the transmission relationship of the epidemic and to identify the source of infection. Additionally, genome sequencing and bioinformatic analysis revealed that the MTB strains from the seven laboratory-confirmed cases in Class 2 belonged to Lineage 2 and were all drug sensitive. Timely and effective interventions prevented the further spread of the TB outbreak. All the cases were eventually treated effectively.

However, this study has some limitations. We inferred from the epidemiological findings that Student A may have been the source of this aggregated school TB outbreak, but do not have molecular biological evidence to support this hypothesis. Follow-up visits were difficult to perform due to school graduation by students with TB. We were unable to confirm whether the 22 students who were strongly positive for TST and received prophylaxis eventually completed the course of treatment. Despite these limitations, this study was able to describe the characteristics and triggers of a large school outbreak in eastern China.

In conclusion, a 2-month delay in diagnosis of the index case played an important role in the transmission of this TB outbreak in a junior high school in Zhejiang, China. The use of Xpert MTB/RIF in BAL samples may improve the sensitivity of laboratory tests and is conducive to investigating the transmission of TB outbreaks. The MTB strains from 7 cases in Class 2 belonged to Lineage 2 and were all drug-sensitive. There is an urgent need to reduce delays in the detection of cases and to isolate the source of infection in time. Efforts should be made to strengthen routine surveillance and health education for students and staff on TB prevention and control in schools.

## Data availability statement

The datasets presented in this study can be found in online repositories. Whole gene sequencing data of strains are deposited in National Microbiology Data Center (NMDC) with accession numbers NMDC40049901 (https://nmdc.cn/resource/genomics/sra/detail/NMDC40049901) and NMDC40049902 (https://nmdc.cn/resource/genomics/sra/detail/NMDC40049902).

## Ethics statement

Written informed consent was obtained from the minor(s)’ legal guardian/next of kin for the publication of any potentially identifiable images or data included in this article.

## Author contributions

QW: Investigation, Software, Writing – original draft. KW: Data curation, Investigation, Software, Writing – review & editing. YZ: Data curation, Investigation, Project administration, Writing – review & editing. ZL: Methodology, Supervision, Writing – review & editing. SC: Methodology, Supervision, Writing – review & editing. XW: Methodology, Supervision, Writing – review & editing. JP: Conceptualization, Methodology, Resources, Supervision, Writing – review & editing. BC: Conceptualization, Funding acquisition, Methodology, Supervision, Writing – review & editing.

## Footnotes

^1^ https://github.com/bcgsc/abyss/tree/bloom-abyss-preview

^2^ https://github.com/lh3/bwa

^3^ http://samtools.sourceforge.net

^4^ https://github.com/amkozlov/raxml-ng
